# Health-related quality of life in young people: the importance of education

**DOI:** 10.1186/s12955-020-01446-5

**Published:** 2020-06-16

**Authors:** Marta Gil-Lacruz, Ana Isabel Gil-Lacruz, María Luisa Gracia-Pérez

**Affiliations:** 1 Department of Psychology and Sociology, Health Science Faculty, Domingo Miral s/n, 50009 Zaragoza, Spain; 2 Department of Management, School of Engineering and Architecture, C. María de Luna, 3, Edificio Betancourt, Campus Río Ebro, 50018 Zaragoza, Spain; 3 Department of Psychology and Sociology, Social and Work Science Faculty, Violante de Hungria 23, 50009 Zaragoza, Spain

## Abstract

**Background:**

The concept of health-related quality of life and education integrates the bio-psychosocial perspective of health and the multidimensional potentialities of education for wellbeing. This present work is especially relevant to young people because understanding the interaction between health and education can facilitate the design of preventive policies. The research examines the way in which the educational level of young people from an urban district in the city of Zaragoza (Casablanca) has an influence on their health-related quality of life (HRQOL).

**Methods:**

A cross sectional survey was undertaken in the Casablanca district of Zaragoza (Spain). Participants were not randomly selected; their numbers reflected the areas where they lived with respect to age and sex distribution. It comprised 122 boys and 122 girls, aged between16 and 29, living in the neighbourhood are: Viñedo Viejo, Las Nieves and Fuentes Claras. These three residence zones are markedly different in terms of socioeconomic composition. The questionnaire included the following information: socioeconomic characteristics (sex, age, educational level, employment status, residence zone), an assessment of health (health problems, diagnosis and medication in the last 2 weeks) and HRQOL (WHOQOL-BREF dimensions: mental health; physical health; social relations; and environment). ANOVA and four regression models were used to assess the role, direction and intensity of educational level on HRQOL.

**Results:**

The results show that the higher the level of education, the better the level of HRQOL. The biggest impact of education was on the mental health dimension, but this influence was modulated by sex and residence zone. The value of the interaction of education and residence zone was more significant than educational level alone. HRQOL of girls is more sensitive to education, being a student and residence zone than the HRQOL of boys.

**Conclusions:**

The dimensions of HRQOL are influenced by educational level. The influence is greatest among girls and the youngest members of the poorest area of the district. Public authorities should contemplate the development of an equitable education system from the beginning of the life cycle as a public health strategy.

## Introduction

Health-related quality of life (HRQOL) is a multidimensional concept that includes domains related to physical, mental, emotional, and social functioning [1]. The development of these domains depends on a set of socioeconomic factors in which educational level has an important role. Education implies learning: knowledge, behaviours, skills and attitudes that can influence health and wellbeing [2, 3].

Improvements in the education system lead to a greater understanding of health issues and better health choices [4, 5]. At the same time, the living conditions and health status of young people (an objective aspect of quality of life) have an impact on their educational and employment possibilities [6, 7]. The World Health Organization [8] believes that good health is both a right of individuals and a resource for societies.

The close relationship between education and health is indisputable at both the individual and public investment level [9]. Health improvements increase access and equity to current educational institutions that are characterised by diversity (students age and ethnical origin, teaching styles, contents and values, curricula requirements, and so on).

Targeted public policies on education may not only have a positive impact on the education level of our young citizens, they could also lead to an improvement on HRQOL. This implies gaining a better understanding of the link between HRQOL and the level of education. This, in turn, requires the consideration of people’s values and experiences related to living conditions, integrating the opinion of those who share the same conditions. This article aims to address this international challenge from a local perspective, the natural context of youth development. The study aims to analyse the relationship between educational level and HRQOL in a sample of urban youth by assessing the impact of socioeconomic variables such as sex, employment status and place of residence.

In a European study of 21,590 children and adolescents, sex was shown to be an important predictor of HRQOL; at a young age both sexes have a similar level, but, with increasing age, the HRQOL of girls is worse than boys [10]. The work by Michel was based on a European sample using the KIDSCREEN-52 questionnaire [10], but the same trend has been reported in studies that have employed other HRQOL instruments, such as the generic EQ-5D [11] and health perceived status instruments like the Short Form Health Survey [12–14]. These differences could be found in diverse indicators for example, stress and accidents are more common among boys and anxiety is more prevalent among girls [15]. In fact, these differences persist through the life span. In the study of Burström et al. [11], HRQOL index was significantly lower in all age-groups for women. Besides, women suffered the greatest increase on anxiety/depression and pain/discomfort (measured with generic EQ-5D, 1998 and 2002 waves).

The exact cause of these health inequalities is open to debate. Socioeconomic variables influence on health differences among young people requests a deeper research [16]. Income, occupation, residence and level of education are still indicators of social class; educational level is related to occupation (access to higher paid, more stable employment) and earnings [17].

Darias [18] listed interactions between educational level and the dimensions of health. First one: Education, family and socioeconomic inheritance span the life cycle and have an impact on the present and future resources of the individual (health status, social networks, living conditions etc.). Second one: A higher educational level allows better access and use of health resources, better understanding of health information and the adoption of healthier lifestyles [19]. Third one: Health problems in childhood are associated with lower academic performance and subsequent health complications that can lead to employment difficulties.

The link among educational level and health with other variables such as sex and age, can adopt an accumulative effect along the life cycle. These interactions lead us to hypothesize that health inequalities increase with age [20].

A variety of theoretical models have aimed to examine the relationship between education and health [21]:

- From a materialistic perspective, the negative consequences of deprivation and social exclusion suggest that a lower level of education translates into worse health indicators; therefore the materialistic perspective includes the social effects of poverty, for example, differences in life expectancy [22, 23].
- In formal education, credentialism (the possession of titles and certificates awarded by the education system) is manifested in better employment opportunities that result in a better quality of life [24]. One of the functions of the education system should be to reduce the health and education gaps that generate the initial social inequalities.
- The influence model of events throughout the life cycle argues that education allows for development of cognitive skills related to personal care [25]. The circumstances in which people live have an obvious impact on health [16].
- The causal selection model focuses on explaining how health complications from childhood are associated with poor academic and work performance. Health is seen as a determining factor for social class - not the other way around [20].

These models of analysis are based on a definition of health that transcends illness and the prevalence of risk behaviours. Educational level is an indicator of social class that compromises the opportunities of personal and social development [17]. The study of HRQOL among young people should not be confined to the field of epidemiology and mental health. The level of health is also impacted by values, representations, beliefs and attitudes [26]. The analysis of the classic indicators of public health (e.g. morbidity, mortality, life expectancy etc.) should be complemented by the study of other factors related to the individual and collective functioning of the person [27, 28]. This scientific literature emphasizes two important dimensions: First one, cognitive, physical and social functioning which refers to the satisfaction generated by positive relationships, the ability to be autonomous, a healthy lifestyle and good mental and physical health. Second one, personal care and emotional wellbeing which include the possibility of learning and acquiring knowledge, the development of personal skills and feelings of happiness and self-esteem, the assessment of physical condition and socioeconomic position [27, 28].

This approach sees the study of HRQOL as a multidimensional concept that integrates physical, psychological and social factors [29]. This concept has been used during the last decade to prioritise medical care needs, measure the degree of wellbeing, and assess the outcomes of clinical treatments [30]. It is also gaining popularity in studies of adolescents and young people, aimed at diagnostic and preventive evaluation [31–34].

In 1991, the World Health Organization [35] led an international project to integrate debates on the multidisciplinary nature of the definition of health-related quality of life. It was intended to emphasise its objective-subjective and individual-social nature and to be contextualised in countries with different levels of industrialisation, health system coverage and cultural diversity (the role of the family, perception of time or religious belief). The term ‘Health-Related Quality of Life’ (HRQOL) was agreed as the individual’s perception of their own vital position, in the context of their culture and value system and in relation to their goals, expectations, standards and interests [36].

This definition is intended to provide a generic measure and contemplates the situation of the person within their reference group [37]. There were six dimensions in the original instrument: physical health; psychological functioning; independence; social relations; environment; and spirituality.

A number of questionnaires inspired by the WHO instrument have been utilised with young people [36, 38]. The longest version (100 items) was used by Cilga [39], who studied the perception of HRQOL among young residents of a vulnerable neighbourhood in Turkey. They found that the physical and psychological functioning of the participants was in need of improvement - 41% of those interviewed said that they had problems coping with pain.

The short version of the WHOQOL (26 items) has also been widely employed. Exponents include projects undertaken in Thailand, New Zealand and China [40–42]. However, some items, such as ‘Live without Pain’ and ‘Confidence in Medication’ were not considered as relevant.

From a comparative and international perspective, age is a source of diversity [43]. Adults prioritise the environment, social support, transportation, the health system and the feeling of being physically fit; younger people give more importance to positive expectations for the future, social relationships and finding an interesting job [43].

The scientific literature focuses on the importance of the relational and environmental dimensions in research on young people and HRQOL [29]. Examples include studies on Ethiopian street youth [44, 45] and student mobbing [46].

As the study of HRQOL involves comparison of reference groups, an open line of research is found in the generalisation of these studies to other types of samples of young people in different socioeconomic and health conditions. A project in Bangladesh [47] analysed the impact of determinants of health such as nutrition and place of residence on the quality of life of adolescents; the results of these types of works have highlighted the impact of contextual variables on the perceived satisfaction of young people [48].

As a context, the school has received considerable attention: HRQOL was directly related to the quality of the educational environment, institutional satisfaction and academic level [48]. Similar results were reported in the university context, where satisfaction with the institution has been directly associated with academic achievement [49]. The feeling of belonging to the educational institution and support offered by the family and peers improves the perception of quality of life [50, 51].

Education seems to have a significant effect on HRQOL; Baumann et al. [52] undertook a study with 355 students from Luxembourg, Belgium and Romania. The psychological dimension of quality of life was positively associated with academic skills and knowledge regarding employability in Luxembourg and Romania, but not in Belgium. It is clear that more research is required in this area.

## Method

This current work focuses on the environment, contextualising the relationship between education and the dimensions of HRQOL in a local community. The main hypothesis is that the higher the level of education, the better the level of HRQOL. However, it is expected that these results will be mediated by quality of life dimensions and sex.

### Sample

Neighbourhoods are the residential units in which young people develop, psychosocial interventions are planned and community participation takes place. The district of Casablanca (Zaragoza, Spain) was chosen for this work due to its socioeconomic diversity and easy access to health resources. Its diversity can be seen in the condition of housing (year of construction, type of construction, facilities such as lifts, gardens etc.) and the socioeconomic characteristics of the residents (occupation, income, age, origin etc.). Furthermore, the urbanisation of the district has resulted in these differences being reflected in three clearly defined areas that are separated by roads, a railway line and a canal.

The three neighbourhoods selected for the study correspond to the socioeconomic stratification of the community: *Fuentes Claras* fits the upper middle-class residential model; *Viñedo Viejo* is the traditional lower-middle/working-class area; and *Las Nieves* is predominantly upper-middle class.

**Fig. 1:**
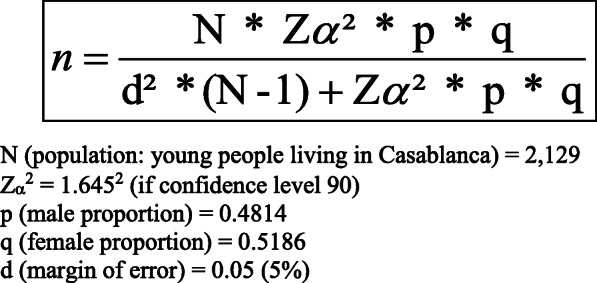
Formula for the sample size determination (n). N (population: young people living in Casablanca) = 2129. Z_α_^2^ = 1.645^2^ (if confidence level 90). p (male proportion) = 0.4814. q (female proportion) = 0.5186. d (margin of error) = 0.05 (5%)

The age range was chosen as many institutions that work with young people (for example, the Spanish Youth Institute) consider ‘young people’ as individuals under the age of 30 and health surveys have a minimum participation age of 16. Assuming a normal distribution of the variables, an α risk of 10%, a δ (error) of 5% and the sex distribution of the population aged 16 to 29 years in Casablanca area, the theoretical sample size was *n* = 240 (Fig. 1). The sample also reflected the socioeconomic distribution of the target population (Table 1).

**Table 1:** Population and sample composition by area of residence and gender

	Casablanca youth population^a^				Sample distribution			
Residence	Males (N)	Females (N)	Total (N)	Total %	Males (*n*)	Females (*n*)	Total (*n*)	Total %
Viñedo Viejo	615	662	1277	60%	79	72	151	62%
Las Nieves	205	221	426	20%	12	28	40	16%
Fuentes Claras	205	221	426	20%	31	22	53	22%
Total	1025	1104	2129	100%	122	122	244	100%

*N* Population size, *n* Sample size

^a^The information provided about sex is the district distribution. The information about residence zone is orientative, because district data are not desegregated at this level. We have used previous studies to calculate it [53]

Participants were recruited at various locations in the district. 17.2% were interviewed in their schools, 4.5% at the Casablanca Youth Centre, 20.9% at their homes and 57.4% in the streets, parks and public places.

Previous to the field research, information about the survey (goals, topics, ethic norms commitment, public funds, researchers contact and so on) was distributed to the neighbourhood by mail boxing. Research team members also visited Casablanca Civic Center, Social Center, Youth Center and the two High Schools of the district. We conducted several interviews with their professionals and directors. All actions were accepted and approved by participants.

Key infomants who collaborate with the survey implementation were: principal researcher and three researchers (Research group: Wellbeing and Social Capital ref. S.51, University of Zaragoza, Spain), two directors of Casablanca Youth Center, the social worker of Health Community Center, three directors of High Schools, five students in training period at Zaragoza University. All these agents shared the same information provided by the principal researcher.

We informed previously to our survey agents about the convenience of keeping the residence zone composition and the population distribution. Two facts were helpful. First one, an important group of young interviewed were alumni of targeted High Schools (located in distant places of the neighbourhood). The source of diversity is important because of the number of interviews and main activity (studying) of the youth at Casablanca. Second one, when training students at Zaragoza University were integrated into the research field, we had information about the number of interviews completed and the need to explore specific zones.

Participation seemed to depend on the location of the interview; for example, in the school and local youth centre, the response rate was 90%, compared with a rate of 70% in public places.

### Instrument

This study is part of a wider research project that was conducted in Casablanca to analyse HRQOL and lifestyles. From the original survey, the statistical exploitation of the following thematic blocks was selected:

- Socioeconomic characteristics: sex, age, marital status, employment status and educational level. Following the criteria of the International Standard Classification of Education [54], educational level was categorised as: no studies; primary; secondary and university (post-secondary education). Educational level was considered as an explanatory variable, the age of the sample meant that all participants could have had secondary or university education. *Secondary* was defined as a dummy variable that informs us if the individual has reached secondary (1) or university (0) education. The main problem with the age group is that many people were too young to have had access to university. For this reason, the category *Student* was introduced, as unemployment in the area is low, working and unemployed people were grouped together as an active population.
- Assessment of health: Health problems, diagnosis by the health service and medication in the last 2 weeks. The wording of these questions was the same as those of the Spanish National Health Survey to allow comparison. The answers to the questions are ‘Yes’ or ‘No’.
- Health-related quality of life: measured by the WHOQOL-BREF [38]. The selection of this instrument was due to its international character and prior use with young people. The instrument was validated by Krägeloh et al. [41] with a sample of students; it was found to have a good level of reliability: Cronbach’s Alpha for the overall scale was 0.89. The criterion-related validity (the correlation of item and domain scores with the score of each of the two global items) and ordinal confirmatory factor analyses also gave positive results*.*
  The questionnaire integrates two generic items about the general perception regarding HRQOL, with four dimensions: physical (7 items; minimum score: 7; maximum score: 35), mental (6 items, minimum score: 6; maximum score: 30), social (3 items, minimum score 3, maximum score 15), and environment (8 items, minimum score 8; maximum score 40). The items have a score from 1 (very negative) to 5 (very positive) on a Likert scale. All the variables are quantitative: higher values imply better health-related quality of life. Further information about scores and psychometric proprieties are provided by Skevington, Lotfy and O’Connell research [38].

### Empirical strategy

The descriptive results of the dependent and explanatory variables were analysed. As dependent variables we have selected the dimensions of WHOQOL-BREF (physical, mental, social, environment), and also their aggregation in one variable. As explanatory variables we considered: sex, age, educational level, employment status, residence zone, and self-assessment of health (this last one, used only on ANOVAs). We have selected three levels of *p* value (< 0.001 < 0.01, < 0.05) in order to explain the significance of the variables in the ANOVAs analysis.

ANOVAs and post hoc Tukey tests were used to identify differences in means of dependent variables among groups created by values of independent variables. Four equation models based on regression analysis were designed to identify differential predictors of HRQOL between girls and boys. Given that the dependent variables are quantitative, Ordinary Least Squares (OLSQ) estimations were used to report data in terms of regression coefficients (STATA command: regress, mfx). OLSQ is a type of linear least squares method for estimating the unknown parameters. Regarding the assumption of linearity, meanwhile our dependent variables are quantitative our explanatory variables are dummy variables, which meet the assumption of linearity by definition, because they create two data points, which define a straight line. The codification of educational level in dummy variables is meaningful and straightforward. Regarding the significance of estimated parameters, these coefficients are useful to indicate how responsive the state of health is to a change in educational level. Independent estimations were calculated for boys and girls to determine if education plays a different role (in terms of sense or intensity) over their health. Four independent models were designed: **Model 1** only considers educational level as an explanatory variable; **Model 2**, takes into account the fact that the participant may still be a student and could reach a higher educational level - the interaction of Secondary*Student (a variable that takes the value 1, if the individual has reached secondary education and is a student, 0 otherwise) has been included; **Model 3** controls for the area of residence as a proxy of social status - the interaction of Secondary*ViñedoViejo (a variable that takes the value 1, if the individual has reached secondary education and lives in Viñedo Viejo, 0 otherwise) has been included; Finally, **Model 4** considers both student status and residence zone - the interactions Secondary*Student and Secondary*Student*ViñedoViejo (a variable that takes the value 1, if the individual has reached secondary education, is a student and lives in Viñedo Viejo and, 0 otherwise) has been included. In OLSQ regressions we have not included neither age nor self-assessment of health as independent variables: age because the estimated coefficients are not statistically significant, so we do not explain them among main results, and self-assessment of health because the estimated coefficients are omitted by collinearity problems. These results are provided in the supplemental material.

Interactions are often considered to control statistically a situation in which there is a simultaneous influence among variables. Is it possible that the educational effect on HRQOL dimensions for young people living in Viñedo Viejo is different than for those living in other residence zones? Or will it be pretty much the same? Interactions answer these questions. In this research we consider the interaction of educational level with employment status and residence zone.

SPSS18.00 and STATA 13.0 were used for statistical analysis.

## Results

The sample was made up of 244 individuals between the ages of 16 and 29 years old- Sample was equally distributed by sex: 122 boys and 122 girls were interviewed. One hundred fifty-one were living at Viñedo Viejo, 40 at Las Nieves and 53 at Fuentes Claras. The mean age of the sample was 21.32 years old (standard deviation = 3.73, mode = 22).

The vast majority of the participants were unmarried (95.9% boys, 94.3% girls) and lived with their parents in four-member households. (Tables 2 and 3 show the main descriptive statistics).

**Table 2:** Descriptive statistics and ANOVAs by health-related quality of life (*n* = 244)

Variables		Physical Health		Mental Health		Relations		Environment	
	N	Mean and SD	Anova *p*-value	Mean and SD	Anova *p*-value	Mean and SD	Anova *p*-value	Mean and SD	Anova *p*-value
**Sex**			0.078		0.002		0.469		0.037
Male	122	28.17 ± 3.33		23.79 ± 2.85		11.87 ± 2.02		30.13 ± 4.04	
Female	122	27.35 ± 3.74		22.55 ± 3.29		11.68 ± 2.09		29.03 ± 4.08	
**Age**			0.653		0.565		0.498		0.097
16 to 18	66	27.63 ± 3,80		22.94 ± 3.36		11.58 ± 2.11		30.39 ± 4.46	
19 to 24	127	27.96 ± 3.38		23.38 ± 3.10		11.92 ± 2.08		29.50 ± 3,94	
25 to 29	51	27.45 ± 3.72		22.96 ± 2.93		11.61 ± 1.91		28.74 ± 3.85	
**Educational level**			0.069		0.003		0.002		0.081
Primary	19	26.32 ± 4.52		22.37 ± 4.15		10.63 ± 2.44		30.17 ± 5.19	
Secondary	118	27.57 ± 3.56		22.60 ± 2.92		11.49 ± 2.03		28.97 ± 4.02	
University	107	28.24 ± 3.30		23.94 ± 3.01		12.25 ± 1,90		30.15 ± 3.90	
**Employment status**			0.044		0.009		0.008		0.020
Autonomous worker	15	27.67 ± 4.11		23.53 ± 3.13		12.60 ± 2.06		29.33 ± 4.38	
Employment	77	27.51 ± 3.50		22.79 ± 2.93		11.63 ± 1.97		28.78 ± 4.04	
Unemployment	13	25.54 ± 3.52		20.77 ± 3.34		10.00 ± 2.48		27.31 ± 4.21	
Student	137	28.21 ± 3.45		23.63 ± 3.12		11.96 ± 1.98		30.32 ± 3.93	
Housewife	2	24.00 ± 4.24		20.50 ± 0.70		11.50 ± 0,70		28.00 ± 7.07	
**Residence zone**			0.198		0.087		0.109		0.001
Viñedo Viejo	151	27.54 ± 3.44		22.86 ± 3.17		11.59 ± 2.14		28.92 ± 3.94	
Las Nieves	40	28.69 ± 3.32		24.05 ± 2.88		12.41 ± 1.75		31.50 ± 4.32	
Fuentes Claras	53	27.69 ± 3.99		23.42 ± 3.12		11.92 ± 1.89		29.98 ± 3.88	
**Health problems**			0.000		0.001		0.570		0.006
Yes	53	25.86 ± 3.36		21.96 ± 3.50		11.63 ± 2.56		28.21 ± 4.22	
Not	191	28.28 ± 3.44		23.52 ± 2.94		11.82 ± 1.88		29.96 ± 3.98

**Table 3:** Descriptive statistics of the dependent and explanatory variables (n = 244)

Variables	Description	Females (Observations = 122)		Males (Observations = 122)	
		Mean	SD	Mean	SD
Dependent variables					
*PhysicalHealth*	Seven items. Likert: 1 = nothing; 5 = strongly agree	27.35	3.7	28.17	3.3
*MentalHealth*	Six items. Likert: 1 = nothing; 5 = strongly agree	22.55	3.3	23.79	2.9
*Relations*	Three items. Likert: 1 = nothing; 5 = strongly agree	11.68	2.1	11.87	2.0
*Environment*	Eight items. Likert: 1 = nothing; 5 = strongly agree	29.03	4.1	30.13	4.0
*Total*	Higher score means better health-related quality of life	88.31	12.6	92.18	13.3
Explanatory variables					
*Secondary*	Dummy variable: 1 if the individual has secondary studies, 0 in case of tertiary studies.	0.56	0.5	0.57	0.5
*Student*	Dummy variable: 1 if the individual is a student, 0 otherwise	0.61	0.5	0.52	0.5
*Secondary&Student*	Dummy variable: 1 if the individual has secondary studies and is a student, 0 otherwise.	0.31	0.5	0.30	0.5
*ViñedoViejo*	Dummy variable: 1 if the individual lives in Viñedo Viejo, 0 otherwise.	0.59	0.5	0.65	0.5
*Secondary&ViñedoViejo*	Dummy variable: 1 if the individual has secondary studies and lives in Viñedo Viejo, 0 otherwise.	0.32	0.5	0.39	0.5
*Secondary&Student&ViñedoViejo*	Dummy variable: 1 if the individual has secondary studies, is a student and lives in Viñedo Viejo, 0 otherwise.	0.14	0.3	0.16	0.4

48% of the sample had achieved secondary studies and 44% of interviewers had achieved university studies. 56% of the sample is studying as a main activity. 61% of the girls and 52% of the boys interviewed are studying. 44% of the boys and 32% of the girls are working.

ANOVAS and post hoc Tukey results were (see Table 2):

- Sex: girls had a more negative perception of the *Physical* (*p* < 0.01), *Mental* (*p* < 0.05), and *Environment* (*p* < 0.05), dimensions of HRQOL than boys.
- Age: there were statistically significant differences regarding perception of *Environment* (*p* < 0.01): older participants gave a more negative evaluation.
- Educational level: this variable has a statistical significant impact on all HRQOL dimensions: *Physical* (*p* < 0.01), *Mental* (*p* < 0.05), Relations (*p* < 0.05), and *Environment* (*p* < 0.01).
- Employment status: there are statistical significant differences among employment status and HRQOL dimensions: *Physical* (*p* < 0.05), *Mental* (*p* < 0.05), Relations (*p* < 0.05), and *Environment* (*p* < 0.05).
- Health problems: there is empirical evidence that young people with health problems reported lower levels of following HRQOL dimensions: *Physical* (*p* < 0.01), *Mental* (*p* < 0.01), and *Environment* (*p* < 0.01).

Table 4 shows regression coefficients of educational level for the state of health of young women living in Casablanca. In **Model 1**, there is empirical evidence that health dimensions are determined by education level: the higher level of education, the better the state of health. *Mentalhealth* is the health dimension most sensitive to educational level, followed by *PhsyicalHealth* and *Relations*. The influence of education on *MentalHealth* is more than double that of *Environment*. When controlled for educational attendance, **Model 2** reveals that having secondary education (rather than university) has a negative impact, with the exception of health as an aggregated measure. In the case of *Relations,* the estimated coefficient remains stable, but it increases for *PhysicalHealth* and *MentalHealth*. *Secondary* is modulated by Secondary*Student for *PhysicalHealth*, *MentalHealth*, and *Environment* but not for *Relations*. For example, in *PhysicalHealth*, girls with secondary education have a 245% worse assessment of physical health than girlswith university education. However, this percentage is reduced by 186% (to 59%) if respondents have secondary education and are still students. **Model 3** shows that the variable *Secondary* loses intensity for all health dimensions and has statistical significance for *PhysicalHealth*. Once again, the estimated value of *Secondary* for *Relations* is the most stable. For *MentalHealth*, *PhysicalHealth* and *Environment* the value of the interaction of education with residence zone is more important than educational level alone. For example, with *PhysicalHealth*, results show that girls with *Secondary* education who live in *ViñedoViejo* value their *PhysicalHealth* as 186% worse than girls with *Secondary* education living other areas. Finally, **Model 4** confirms that in the study of the impact of education on the health of girl population groups, it is important to control for student status and residence zone.

**Table 4:** Regression coefficients of educational level among young women living in Casablanca (*n* = 122)

	*PhysicalHealth*		*MentalHealth*		*Relations*		*Environment*		Total	
	Regression coefficients	*p*-value	Regression coefficients	*p*-value	Regression coefficients	Regression coefficients	*p*-value	Regression coefficients	*p*-value	Regression coefficients
**Model 1**										
*Secondary*	−1.463	0.03	−2.165	0.00	−1.235	0.00	−1.017	0.17	−8.563	0.00
**Model 2**										
*Secondary*	−2.451	0.00	−2.607	0.00	−1.221	0.01	−1.993	0.030	−7.522	0.01
*Secondary& Student*	1.859	0.04	0.811	0.29	−0.029	0.96	1.789	0.073	−1.885	0.52
**Model 3**										
*Secondary*	−0.373	0.67	−1.276	0.08	−1.035	0.05	0.649	0.47	−7.090	0.01
*Secondary& ViñedoViejo*	−1.886	0.04	−1.543	0.05	−0.313	0.57	−2.971	0.00	−2.597	0.38
**Model 4**										
*Secondary*	−2.451	0.00	−2.607	0.00	−1.221	0.01	−1.993	0.03	−7.522	0.01
*Secondary& Student*	2.826	0.01	1.717	0.05	0.131	0.85	3.210	0.00	−0.200	0.95
*Secondary& Student& ViñedoViejo*	−2.193	0.08	−2.037	0.05	−0.297	0.70	−3.410	0.01	−3.896	0.33

Table 5 gives the regression coefficients of educational level for the state of health of boys living in Casablanca. The first notable result is that in **Model 1** there is no evidence of the impact of education on the state of health of boys. However, the situation changes with **Model 2** which shows that boys with *Secondary* education have lower levels of *MentalHealth*, *Relations*, *Environment* than men with university education, although this influence disappears for *MentalHealth* and *Environment* when the boys are students, in other words, the negative influence of having secondary education compared to university on health only remains for boys with secondary education who have already left the educational system. **Model 3** does not provide any robust empirical evidence for boys. **Model 4** confirms the results obtained in **Model 2** for the *MentalHealth* and *Environment*.

**Table 5:** Regression coefficients of educational level among young men living in Casablanca (*n* = 122)

	*PhysicalHealth*		*MentalHealth*		*Relations*		*Environment*		Total	
	Regression coefficients	*p*-value	Regression coefficients	*p*-value	Regression coefficients	*p*-value	Regression coefficients	*p*-value	Regression coefficients	*p*-value
**Model 1**										
*Secondary*	−0.290	0.64	−0.632	0.23	−0.539	0.15	−1.059	0.16	−1.950	0.42
**Model 2**										
*Secondary*	−1.052	0.16	−1.623	0.01	−0.912	0.04	−2.505	0.01	−5.221	0.08
*Secondary& Student*	1.421	0.08	1.847	0.01	0.713	0.15	2.691	0.01	6.100	0.06
**Model 3**										
*Secondary*	−0.190	0.83	−0.297	0.69	−0.328	0.54	−0.831	0.44	−2.093	0.54
*Secondary& ViñedoViejo*	−0.143	0.87	−0.482	0.52	−0.299	0.59	−0.326	0.76	0.205	0.95
**Model 4**										
*Secondary*	−1.052	0.16	−1.623	0.01	−0.912	0.05	−2.505	0.01	−5.221	0.08
*Secondary& Student*	1.219	0.21	1.858	0.02	0.617	0.32	2.833	0.02	4.715	0.23
*Secondary& Student& ViñedoViejo*	0.395	0.72	−0.020	0.98	0.181	0.79	−0.269	0.84	2.696	0.54

**Fig. 2:**
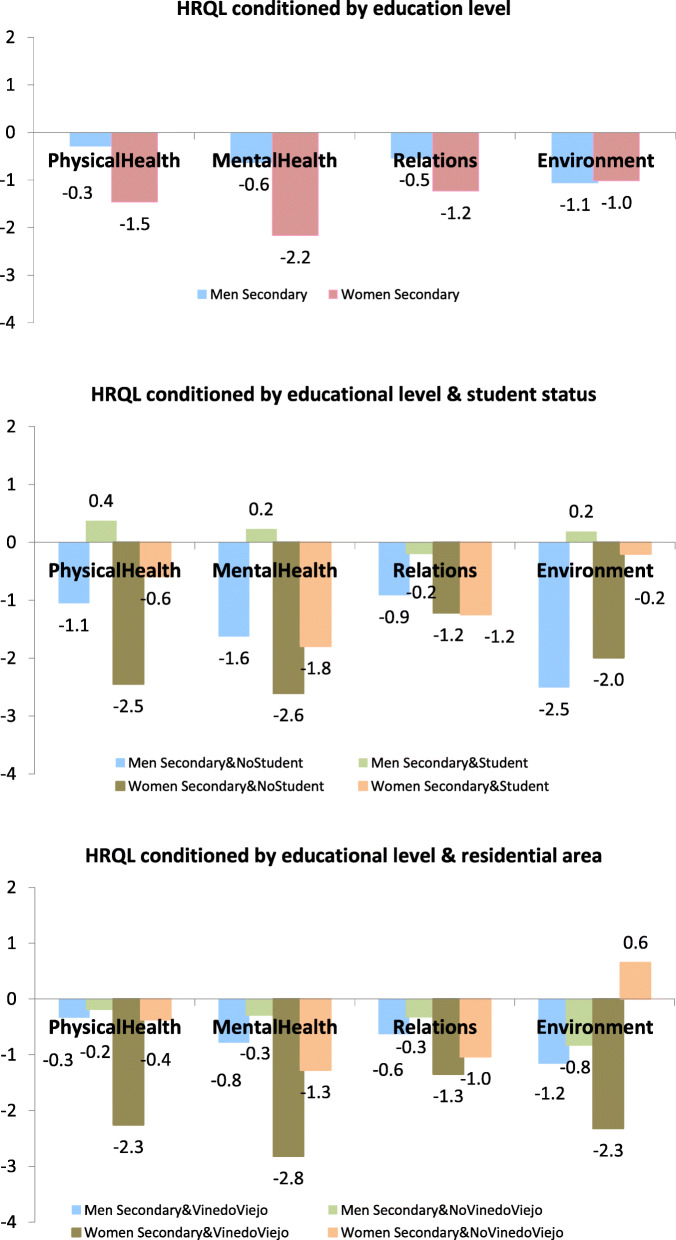
Educational effects on HRQL dimensions

Figure 2 is a compilation of previous results (Tables 4 and 5) illustrating visually the impact of education over health (**Model 1**) and how it is modulated when controlled by educational status (**Model 2**) and residence zone (**Model 3**). Each legend shows the educational impact controlling by the additional characteristics included in the legend, thus for example, the legend *MenSecondary* shows that boys with secondary education report a worst state of health for each category in relation to boys with university education, being the estimated coefficient the difference among both groups. Following this argumentation line *MenSecondary&NoStudent* and *MenSecondary&Student* control prior results depending if the boys are still student or not. Then *MenSecondary&NoStudent* report the additional impact of secondary versus university education for boys with secondary education that are no longer students. Gender differences reveal that girls ‘state of health is more sensitive to education, student status and residence zone compared to boys.

## Discussion

The results of this study show a clear relationship between a higher educational level and a higher level of HRQOL. This can be interpreted by means of materialistic theories that argue that the objective indicators of social class are also reflected in self-assessment and health indicators [21].

Although, in general, university students in the sample seem to enjoy a better health-related quality of life, this association is characterised by the fact that the sample was heterogeneous. 65% of the boys lived in Viñedo Viejo, compared to 59% of the girls and this might explain the differences observed in the student status. Distribution by educational level and residence zone was comparable: 54% of boys and 60% of girls who lived in Viñedo Viejo had secondary education but this result changes when student status is considered: just 43% of boys and 41% of girls who live in Viñedo Viejo and have secondary studies are still students - 10% less than the average percentage for Casablanca as a whole.

Respondents with secondary education were those who gave a worse evaluation of the health of their environment. The age of the respondents may influence trends: the sample age range encompassed adolescence and youth, with their corresponding vital challenges. This finding is coherent with WHOQOL-BREF research by Saxena et al. [43] which compared adults and young people: adults prioritise environment and physical function; younger people prioritise their social domain.

Sex is a significant variable; it can be inferred that the level of education generates more differences among girls than boys. HRQOL was more sensitive to changes in educational level among girls than boys. These results confirm that women report lower states of health, but this may be due to the influence of factors related to gender roles - men tend to complain less about their ailments and make less use of health services [20]. Differential gender attitudes of teachers, health professionals, parents and other social agents may also have an important influence. Gidley [55] argues that it may be the case that the education system is part of the inequity problem but it is definitely part of the solution: equity, pluralism and respect are values that can be taught and learnt in class.

The educational level had more specific weight in the four dimensions of HRQOL for girls. This was most notable for mental health [21]. It could be inferred that university education is a protective factor in the perceived quality of life of girls.

The fact that educational level, student status and residence zone have a joint influence on HRQOL of both boys and girls leads us to the stratification process. Among girls, the Physical and Environment dimensions were more important than Social and Residence (more than the educational level by itself). For boys, this interaction was evident in the Mental and Environment dimensions.

The results indicate that gender remains a significant source of inequality that should be taken into account in both the training of educational agents and health professionals [56]. It is a variable that influences identity, personal care and the use of health resources [57]. The persistence of significant statistical differences between boys and girls from Casablanca suggests that role models are still important [53].

### Limitations and strengths

Age is a variable which may interact with educational level as the youngest participants of our sample are still school students therefore they have not had the chance to attend university. This is an issue that requires more in-depth research and a cohort perspective to analyze.

The study is cross-sectional and does not therefore consider the evolution of inequity. A longitudinal approach could provide information on the continuity of differences. In the same way, because this community research was conducted previous to the international economic crisis of 2007, an interesting future line of study is replying the study now, in order to capture tendencies and to measure the impact of this crisis.

The data is quantitative, research on perceptions, motivations or the evaluation of quality of life could be complemented by a qualitative methodology.

The main strength of this study is its local context. In addition to the use of national and international databases, information has been drawn from a specific community. The neighbourhood, as a place of residence and shared space, is the territorial unit closest to the configuration of healthy spaces. The results of the analysis can be used to generate programmes focused on the needs of the community, based on the principles of primary care and the promotion of citizen participation in education and health.

New lines of research arise from this work: young people grow up and develop in a family socioeconomic situation so it would be interesting to study how the level of education of the parents affects family lifestyles; the degree of aspiration and issues of gender differences are also of interest, the encouragement that parents, regardless of their level of education, give to their children to study and the extent to which they direct them towards learning and academic achievement.

## Conclusions

A discussion on HRQOL and its association with education implies contemplation of the role of educational agents and instruction on lifestyles.

The results of this study illustrate the need for a set of social policies that:

- Increase equality of opportunities to access to higher education: there should be more scholarships and grants for students in unfavourable socioeconomic situations
- Improve informal education: the knowledge and skills acquired in non-academic contexts; in general, low socioeconomic contexts offer less health education and this increases exposure to risk factors. These inequalities should be addressed through prevention and awareness campaigns.
- Target young people and focus on health and education [58]. Health knowledge and the teaching of skills to minimise exposure to risk factors are of particular importance [28].

From a local and community perspective, the Casablanca Youth Centre, the Health Centre, schools and other institutions that are responsible for health and education in the community, could jointly instigate preventative instruction programmes for children and adolescents. In Spain, the Health Community Councils and Education Community Councils are legally responsible for this work which includes programmes on alcohol and drug abuse, sex education and road traffic safety. It also is clear that more attention must be paid to gender issues concerning the interactions between health perception, quality of life and wellbeing.

The results obtained in Casablanca suggest that it is necessary to consider the scope of intervention of local policies. The neighbourhood is the context where citizen participation in public health management can be fostered. Policies should aim to empower citizens by developing the role of health councils as the foundation of primary health care. The local environment favours direct participation: it is a relatively small space in which the institutions closest to young people are integrated - families, groups of friends, schools, health centres etc. [59].

## Supplementary information

**Additional file 1: Table S1.** Regression coefficients of educational level among young women living in Casablanca (*n* = 122): models for physical health. **Table S2.** Regression coefficients of educational level among young women living in Casablanca (*n* = 122): models for mental health. **Table S3.** Regression coefficients of educational level among young women living in Casablanca (*n* = 122): models for relations. **Table S4.** Regression coefficients of educational level among young women living in Casablanca (*n* = 122): models for environment. **Table S5.** Regression coefficients of educational level among young women living in Casablanca (*n* = 122): models for total health. **Table S6.** Regression coefficients of educational level among young men living in Casablanca (*n* = 122): models for physical health. **Table S7.** Regression coefficients of educational level among young men living in Casablanca (*n* = 122): models for mental health. **Table S8.** Regression coefficients of educational level among young men living in Casablanca (*n* = 122): models for relations. **Table S9.** Regression coefficients of educational level among young men living in Casablanca (*n* = 122): models for enviroment. **Table S10.** Regression coefficients of educational level among young men living in Casablanca (*n* = 122): models for total.

## Data Availability

Please contact author for data request.

## Abbreviations

EQ-5D: EuroQol five dimensions scale
HRQOL: Health related quality of life
ISCED: International Standard Classification of Education
OLS: Ordinary Least Squares
SPSS: Statistical package for social sciences
STATA: Data analysis and statistical software
WHO: World Health Organization
WHOQOL: World Health Organization quality of life
WHOQOL-BREF: World Health Organization quality of life BREF test
