# Supplementary material for: Health-related quality of life in young people: the importance of education
Source: Health Qual Life Outcomes. 2020 Jun 16;18:187. doi: 10.1186/s12955-020-01446-5 (PMC7298764; doi:10.1186/s12955-020-01446-5)
Supplement: Supplementary file 1 — Additional file 1: Table S1. Regression coefficients of educational level among young women living in Casablanca (n = 122): models for physical health. Table S2. Regression coefficients of educational level among young women living in Casablanca (n = 122): models for mental health. Table S3. Regression coefficients of educational level among young women living in Casablanca (n = 122): models for relations. Table S4. Regression coefficients of educational level among young women living in Casablanca (n = 122): models for environment. Table S5. Regression coefficients of educational level among young women living in Casablanca (n = 122): models for total health. Table S6. Regression coefficients of educational level among young men living in Casablanca (n = 122): models for physical health. Table S7. Regression coefficients of educational level among young men living in Casablanca (n = 122): models for mental health. Table S8. Regression coefficients of educational level among young men living in Casablanca (n = 122): models for relations. Table S9. Regression coefficients of educational level among young men living in Casablanca (n = 122): models for enviroment. Table S10. Regression coefficients of educational level among young men living in Casablanca (n = 122): models for total. [file 12955_2020_1446_MOESM1_ESM.docx]

Table S1: Regression coefficients of educational level among young women living in Casablanca (n=122): models for physical health

| Explanatory variables | Prob | Initial model | | Final model | |
| --- | --- | --- | --- | --- | --- |
|  |  | Regression coefficient | p-value | Regression coefficient | p-value |
| **Model 1** | | | | | |
| *Secondary No Yes* | 45%  55% | 0.000  -2.021 | --  0.01 | 0.000  -1.463 | --  0.03 |
| *Age 16-18  19-24  25-29* | 26%  72%  2% | 1.284  0.000  1.418 | 0.14  --  0.59 |  |  |
| *Health No problems Yes* | --  -- | --  -- | (omitted because of collinearity) |  |  |
| **Model 2** | | | | | |
| *Secondary No  Yes* | 45%  55% | 0.000  -2.598 | --  0.00 | 0.000  -2.451 | --  0.00 |
| *Secondary & No Student Yes* | 71%  29% | 0.000  1.818 | --  0.11 | 0.000  1.859 | --  0.04 |
| *Age 16-18  19-24  25-29* | 26%  72%  2% | 0.268  0.000  1.957 | 0.80  --  0.46 |  |  |
| *Health No problems Yes* | --  -- | --  -- | (omitted because of collinearity) |  |  |
| **Model 3** | | | | | |
| *Secondary No  Yes* | 45%  55% | 0.000  -0.780 | --  0.47 | 0.000  -0.373 | --  0.67 |
| *Secondary & No ViñedoViejo Yes* | 68%  32% | 0.000  -1.704 | --  0.09 | 0.000  -1.886 | --  0.04 |
| *Age 16-18  19-24  25-29* | 26%  72%  2% | 0.612  0.000  1.857 | 0.52  --  0.48 |  |  |
| *Health No problems Yes* | --  -- | --  -- | (omitted because of collinearity) |  |  |
| **Model 4** |  |  |  |  |  |
| *Secondary No  Yes* | 45%  55% | 0.000  -2.535 | --  0.003 | 0.000  -2.451 | --  0.00 |
| *Secondary & No Student Yes* | 71%  29% | 0.000  3.481 | --  0.018 | 0.000  2.826 | --  0.01 |
| *Secondary & Student & No ViñedoViejo Yes* | 87%  13% | 0.000  -2.494 | --  0.070 | 0.000  -2.193 | --  0.08 |
| *Age 16-18  19-24  25-29* | 26%  72%  2% | -0.627  0.000  1.861 | 0.598  --  0.484 |  |  |
| *Health No problems Yes* | --  -- | --  -- | (omitted because of collinearity) |  |  |

Table S2: Regression coefficients of educational level among young women living in Casablanca (n=122): models for mental health

| Explanatory variables | Prob | Initial model | | Final model | |
| --- | --- | --- | --- | --- | --- |
|  |  | Regression coefficient | p-value | Regression coefficient | p-value |
| **Model 1** | | | | | |
| *Secondary No  Yes* | 45%  55% | 0.000  -2.020 | --  0.01 | 0.000  -2.165 | --  0.00 |
| *Age 16-18  19-24  25-29* | 26%  72%  2% | 1.283  0.000  1.418 | 0.14  --  0.59 |  |  |
| *Health No problems Yes* | --  -- | --  -- | (omitted because of collinearity) |  |  |
| **Model 2** | | | | | |
| *Secondary No  Yes* | 45%  55% | 0.000  -2.598 | --  0.00 | 0.000  -2.607 | --  0.00 |
| *Secondary & No Student Yes* | 71%  29% | 0.000  1.818 | --  0.11 | 0.00  0.811 | --  0.29 |
| *Age 16-18  19-24  25-29* | 26%  72%  2% | 0.268  0.00  1.957 | 0.80  --  0.46 |  |  |
| *Health No problems Yes* | --  -- | --  -- | (omitted because of collinearity) |  |  |
| **Model 3** | | | | | |
| *Secondary No  Yes* | 45%  55% | 0.000  -0.780 | --  0.47 | 0.000  -1.276 | --  0.08 |
| *Secondary & No ViñedoViejo Yes* | 68%  32% | 0.000  -1.704 | --  0.09 | 0.000  -1.543 | --  0.05 |
| *Age 16-18  19-24  25-29* | 26%  72%  2% | 0.612  0.000  1.857 | 0.52  --  0.48 |  |  |
| *Health No problems Yes* | --  -- | --  -- | (omitted because of collinearity) |  |  |
| **Model 4** |  |  |  |  |  |
| *Secondary No  Yes* | 45%  55% | 0.000  -2.536 | --  0.00 | 0.000  -2.607 | --  0.00 |
| *Secondary & No Student Yes* | 71%  29% | 0.000  3.481 | --  0.01 | 0.000  1.717 | --  0.05 |
| *Secondary & Student & No ViñedoViejo Yes* | 87%  13% | 0.000  -2.494 | --  0.07 | 0.000  -2.037 | --  0.05 |
| *Age 16-18  19-24  25-29* | 26%  72%  2% | -0.627  0.000  1.861 | 0.59  --  0.48 |  |  |
| *Health No problems Yes* | --  -- | --  -- | (omitted because of collinearity) |  |  |

Table S3: Regression coefficients of educational level among young women living in Casablanca (n=122): models for relations

| Explanatory variables | Prob | Initial model | | Final model | |
| --- | --- | --- | --- | --- | --- |
|  |  | Regression coefficient | p-value | Regression coefficient | p-value |
| **Model 1** | | | | | |
| *Secondary No  Yes* | 45%  55% | -1.302 | 0.00 | 0.000  -1.235 | --  0.00 |
| *Age 16-18  19-24  25-29* | 26%  72%  2% | -0.002  0.000  1.981 | 0.99  --  0.17 |  |  |
| *Health No problems Yes* | --  -- | --  -- | (omitted because of collinearity) |  |  |
| **Model 2** | | | | | |
| *Secondary No  Yes* | 45%  55% | 0.000  -1.350 | --  0.00 | 0.000  -1.221 | --  0.01 |
| *Secondary & No Student Yes* | 71%  29% | 0.000  0.154 | --  0.81 | 0.000  -0.029 | --  0.96 |
| *Age 16-18  19-24  25-29* | 26%  72%  2% | -0.083  0.000  2.026 | 0.89  0.17 |  |  |
| *Health No problems Yes* | --  -- | --  -- | (omitted because of collinearity) |  |  |
| **Model 3** | | | | | |
| *Secondary No  Yes* | 45%  55% | 0.000  -0.946 | --  0.12 | 0.000  -1.035 | --  0.05 |
| *Secondary & No ViñedoViejo Yes* | 68%  32% | 0.000  -0.483 | --  0.41 | 0.000  -0.313 | --  0.57 |
| *Age 16-18  19-24  25-29* | 26%  72%  2% | -.152  0.000  2.103 | 0.78  --  0.15 |  |  |
| *Health No problems Yes* | --  -- | --  -- | (omitted because of collinearity) |  |  |
| **Model 4** |  |  |  |  |  |
| *Secondary No  Yes* | 45%  55% | 0.000  -1.342 | --  0.00 | 0.000  -1.221 | --  0.01 |
| *Secondary & No Student Yes* | 71%  29% | 0.000  0.428 | --  0.62 | 0.000  0.131 | --  0.85 |
| *Secondary & Student & No ViñedoViejo Yes* | 87%  13% | 0.000  -0.387 | --  0.63 | 0.000  -0.297 | --  0.70 |
| *Age 16-18  19-24  25-29* | 26%  72%  2% | -0.198  0.000  2.014 | 0.76  --  0.17 |  |  |
| *Health No problems Yes* | --  -- | --  -- | (omitted because of collinearity) |  |  |

Table S4: Regression coefficients of educational level among young women living in Casablanca (n=122): models for environment

| Explanatory variables | Prob | Initial model | | Final model | |
| --- | --- | --- | --- | --- | --- |
|  |  | Regression coefficient | p-value | Regression coefficient | p-value |
| **Model 1** | | | | | |
| *Secondary No  Yes* | 45%  55% | 0.000  -1.863 | --  0.03 | 0.000  -1.017 | --  0.17 |
| *Age 16-18  19-24  25-29* | 26%  72%  2% | 1.863  0.000  1.340 | 0.05  --  0.64 |  |  |
| *Health No problems Yes* | --  -- | --  -- | (omitted because of collinearity) |  |  |
| **Model 2** | | | | | |
| *Secondary No  Yes* | 45%  55% | 0.000  -2.179 | --  0.02 | 0.000  -1.993 | --  0.03 |
| *Secondary & No Student Yes* | 71%  29% | 0.000  1.057 | --  0.42 | 0.000  1.789 | --  0.07 |
| *Age 16-18  19-24  25-29* | 26%  72%  2% | 1.240  0.000  1.632 | 0.31  --  0.58 |  |  |
| *Health No problems Yes* | --  -- | --  -- | (omitted because of collinearity) |  |  |
| **Model 3** | | | | | |
| *Secondary No  Yes* | 45%  55% | 0.000  0.092 | --  0.93 | 0.000  0.649 | --  0.47 |
| *Secondary & No ViñedoViejo Yes* | 68%  32% | 0.000  -2.715 | --  0.01 | 0.000  -2.971 | --  0.00 |
| *Age 16-18  19-24  25-29* | 26%  72%  2% | 0.809  0.000  2.059 | 0.42  --  0.47 |  |  |
| *Health No problems Yes* | --  -- | --  -- | (omitted because of collinearity) |  |  |
| **Model 4** |  |  |  |  |  |
| *Secondary No  Yes* | 45%  55% | 0.000  -2.099 | --  0.02 | 0.000  -1.993 | --  0.03 |
| *Secondary & No Student Yes* | 71%  29% | 0.000  3.225 | --  0.04 | 0.000  3.210 | --  0.00 |
| *Secondary & Student & No ViñedoViejo Yes* | 87%  13% | 0.000  -3.367 | --  0.02 | 0.000  -3.410 | --  0.01 |
| *Age 16-18  19-24  25-29* | 26%  72%  2% | 0.099  0.000  1.510 | 0.93  --  0.60 |  |  |
| *Health No problems Yes* | --  -- | --  -- | (omitted because of collinearity) |  |  |

Table S5: Regression coefficients of educational level among young women living in Casablanca (n=122): models for total health

| Explanatory variables | Prob | Initial model | | Final model | |
| --- | --- | --- | --- | --- | --- |
|  |  | Regression coefficient | p-value | Regression coefficient | p-value |
| **Model 1** | | | | | |
| *Secondary No  Yes* | 45%  55% | 0.000  -8.236 | --  0.00 | 0.000  -8.563 | --  0.00 |
| *Age 16-18  19-24  25-29* | 26%  72%  2% | -1.337  0.000  8.131 | 0.63  --  0.34 |  |  |
| *Health No problems Yes* | --  -- | --  -- | (omitted because of collinearity) |  |  |
| **Model 2** | | | | | |
| *Secondary No  Yes* | 45%  55% | 0.000  -7.992 | --  0.00 | 0.000  -7.522 | --  0.01 |
| *Secondary & No Student Yes* | 71%  29% | 0.000  -0.766 | --  0.84 | 0.000  -1.885 | --  0.52 |
| *Age 16-18  19-24  25-29* | 26%  72%  2% | -0.899  0.000  7.903 | 0.80  --  0.36 |  |  |
| *Health No problems Yes* | --  -- | --  -- | (omitted because of collinearity) |  |  |
| **Model 3** | | | | | |
| *Secondary No  Yes* | 45%  55% | 0.000  -4.941 | --  0.15 | 0.000  -7.090 | --  0.01 |
| *Secondary & No ViñedoViejo Yes* | 68%  32% | 0.000  -4.527 | --  0.16 | 0.000  -2.597 | --  0.38 |
| *Age 16-18  19-24  25-29* | 26%  72%  2% | -3.126  0.000  9.298 | 0.31  --  0.28 |  |  |
| *Health No problems Yes* | --  -- | --  -- | (omitted because of collinearity) |  |  |
| **Model 4** |  |  |  |  |  |
| *Secondary No  Yes* | 45%  55% | 0.000  -7.862 | --  0.00 | 0.000  -7.522 | --  0.01 |
| *Secondary & No Student Yes* | 71%  29% | 0.000  2.657 | --  0.57 | 0.000  -0.200 | --  0.95 |
| *Secondary & Student & No ViñedoViejo Yes* | 87%  13% | 0.000  -5.139 | --  0.23 | 0.000  -3.896 | --  0.33 |
| *Age 16-18  19-24  25-29* | 26%  72%  2% | -2.749  0.000  7.705 | 0.47  --  0.38 |  |  |
| *Health No problems Yes* | --  -- | --  -- | (omitted because of collinearity) |  |  |

Table S6: Regression coefficients of educational level among young men living in Casablanca (n=122): models for physical health

| Explanatory variables | Prob | Initial model | | Final model | |
| --- | --- | --- | --- | --- | --- |
|  |  | Regression coefficient | p-value | Regression coefficient | p-value |
| **Model 1** | | | | | |
| *Secondary No  Yes* | 43%  57% | 0.000  0.004 | --  0.99 | 0.000  -0.290 | --  0.64 |
| *Age 16-18  19-24  25-29* | 27%  67%  6% | 0.618  0.000  -0.655 | 0.45  --  0.06 |  |  |
| *Health No problems Yes* | --  -- | --  -- | (omitted because of collinearity) |  |  |
| **Model 2** | | | | | |
| *Secondary No  Yes* | 43%  57% | 0.000  0.718 | --  0.34 | 0.000  -1.052 | --  0.16 |
| *Secondary & No Student Yes* | 69%  31% | 0.000  2.564 | --  0.01 | 0.000  1.421 | --  0.08 |
| *Age 16-18  19-24  25-29* | 27%  67%  6% | -2.042  0.000  -0.221 | 0.03  --  0.86 |  |  |
| *Health No problems Yes* | --  -- | --  -- | (omitted because of collinearity) |  |  |
| **Model 3** | | | | | |
| *Secondary No  Yes* | 43%  57% | 0.000  0.357 | --  0.74 | 0.000  -0.190 | --  0.83 |
| *Secondary & No ViñedoViejo Yes* | 60%  40% | 0.000  -0.420 | --  0.66 | 0.000  -0.143 | --  0.87 |
| *Age 16-18  19-24  25-29* | 27%  67%  6% | -0.767  0.000  -0.619 | 0.39  --  0.644 |  |  |
| *Health No problems Yes* | --  -- | --  -- | (omitted because of collinearity) |  |  |
| **Model 4** |  |  |  |  |  |
| *Secondary No  Yes* | 43%  57% | 0.000  -0.717 | --  0.34 | 0.000  -1.052 | --  0.16 |
| *Secondary & No Student Yes* | 69%  31% | 0.000  2.575 | --  0.03 | 0.000  1.219 | --  0.21 |
| *Secondary & Student & No ViñedoViejo Yes* | 84%  16% | 0.000  -0.017 | --  0.98 | 0.000  0.395 | --  0.72 |
| *Age 16-18  19-24  25-29* | 27%  67%  6% | -2.045  0.000  -0.221 | 0.03  --  0.86 |  |  |
| *Health No problems Yes* | --  -- | --  -- | (omitted because of collinearity) |  |  |

Table S7: Regression coefficients of educational level among young men living in Casablanca (n=122): models for mental health

| Explanatory variables | Prob | Initial model | | Final model | |
| --- | --- | --- | --- | --- | --- |
|  |  | Regression coefficient | p-value | Regression coefficient | p-value |
| **Model 1** | | | | | |
| *Secondary No  Yes* | 43%  57% | 0.000  -0.978 | --  0.11 | 0.000  -0.632 | --  0.23 |
| *Age 16-18  19-24  25-29* | 27%  67%  6% | 0.747  0.000  0.278 | 0.28  --  0.80 |  |  |
| *Health No problems Yes* | --  -- | --  -- | (omitted because of collinearity) |  |  |
| **Model 2** | | | | | |
| *Secondary No  Yes* | 43%  57% | 0.000  -1.592 | --  0.01 | 0.000  -1.623 | --  0.01 |
| *Secondary & No Student Yes* | 69%  31% | 0.000  2.200 | --  0.00 | 0.000  1.847 | --  0.01 |
| *Age 16-18  19-24  25-29* | 27%  67%  6% | -0.474  0.000  0.650 | 0.56  --  0.55 |  |  |
| *Health No problems Yes* | --  -- | --  -- | (omitted because of collinearity) |  |  |
| **Model 3** | | | | | |
| *Secondary No  Yes* | 43%  57% | 0.000  -0.789 | --  0.40 | 0.000  -0.297 | --  0.69 |
| *Secondary & No ViñedoViejo Yes* | 60%  40% | 0.000  -0.220 | --  0.78 | 0.000  -0.482 | --  0.52 |
| *Age 16-18  19-24  25-29* | 27%  67%  6% | 0.668  0.297 | 0.37  --  0.79 |  |  |
| *Health No problems Yes* | --  -- | --  -- | (omitted because of collinearity) |  |  |
| **Model 4** |  |  |  |  |  |
| *Secondary No  Yes* | 43%  57% | 0.000  -1.589 | --  0.01 | 0.000  -1.623 | --  0.01 |
| *Secondary & No Student Yes* | 69%  31% | 0.000  2.273 | --  0.02 | 0.000  1.858 | --  0.02 |
| *Secondary & Student & No ViñedoViejo Yes* | 84%  16% | 0.000  -0.120 | --  0.89 | 0.000  -0.020 | --  0.98 |
| *Age 16-18  19-24  25-29* | 27%  67%  6% | -0.494  0.000  0.648 | 0.55  --  0.56 |  |  |
| *Health No problems Yes* | --  -- | --  -- | (omitted because of collinearity) |  |  |

Table S8: Regression coefficients of educational level among young men living in Casablanca (n=122): models for relations

| Explanatory variables | Prob | Initial model | | Final model | |
| --- | --- | --- | --- | --- | --- |
|  |  | Regression coefficient | p-value | Regression coefficient | p-value |
| **Model 1** | | | | | |
| *Secondary No  Yes* | 43%  57% | 0.000  -0.791 | --  0.06 | 0.000  -0.539 | --  0.15 |
| *Age 16-18  19-24  25-29* | 27%  67%  6% | 0.550  0.000  -0.643 | 0.27  --  0.45 |  |  |
| *Health No problems Yes* | --  -- | --  -- | (omitted because of collinearity) |  |  |
| **Model 2** | | | | | |
| *Secondary No  Yes* | 43%  57% | 0.000  -0.933 | --  0.04 | 0.000  -0.912 | --  0.04 |
| *Secondary & No Student Yes* | 69%  31% | 0.000  0.505 | --  0.40 | 0.000  0.713 | --  0.15 |
| *Age 16-18  19-24  25-29* | 27%  67%  6% | 0.279  0.000  -0.568 | 0.64  --  0.50 |  |  |
| *Health No problems Yes* | --  -- | --  -- | (omitted because of collinearity) |  |  |
| **Model 3** | | | | | |
| *Secondary No  Yes* | 43%  57% | 0.000  -0.760 | --  0.25 | 0.000  -0.328 | --  0.54 |
| *Secondary & No ViñedoViejo Yes* | 60%  40% | 0.000  -0.035 | --  0.95 | 0.000  -0.299 | 0.59 |
| *Age 16-18  19-24  25-29* | 27%  67%  6% | 0.538  0.000  -0.641 | 0.32  --  0.45 |  |  |
| *Health No problems Yes* | --  -- | --  -- | (omitted because of collinearity) |  |  |
| **Model 4** |  |  |  |  |  |
| *Secondary No  Yes* | 43%  57% | 0.000  -0.940 | --  0.04 | 0.000  -0.912 | --  0.05 |
| *Secondary & No Student Yes* | 69%  31% | 0.000  0.356 | --  0.63 | 0.000  0.617 | --  0.32 |
| *Secondary & Student & No ViñedoViejo Yes* | 84%  16% | 0.000  0.244 | --  0.73 | 0.000  0.181 | --  0.79 |
| *Age 16-18  19-24  25-29* | 27%  67%  6% | 0.316  0.000  -0.565 | 0.60  --  0.51 |  |  |
| *Health No problems Yes* | --  -- | --  -- | (omitted because of collinearity) |  |  |

Table S9: Regression coefficients of educational level among young men living in Casablanca (n=122): models for enviroment

| Explanatory variables | Prob | Initial model | | Final model | |
| --- | --- | --- | --- | --- | --- |
|  |  | Regression coefficient | p-value | Regression coefficient | p-value |
| **Model 1** | | | | | |
| *Secondary No  Yes* | 43%  57% | 0.000  -2.400 | --  0.00 | 0.000  -1.059 | --  0.16 |
| *Age 16-18  19-24  25-29* | 27%  67%  6% | 2.932  0.000  1.205 | 0.00  --  0.46 |  |  |
| *Health No problems Yes* | --  -- | --  -- | (omitted because of collinearity) |  |  |
| **Model 2** | | | | | |
| *Secondary No  Yes* | 43%  57% | 0.000  -2.883 | --  0.00 | 0.000  -2.505 | --  0.01 |
| *Secondary & No Student Yes* | 69%  31% | 0.000  1.714 | --  0.13 | 0.000  2.691 | --  0.01 |
| *Age 16-18  19-24  25-29* | 27%  67%  6% | 1.992  0.000  1.461 | 0.08  --  0.37 |  |  |
| *Health No problems Yes* | --  -- | --  -- | (omitted because of collinearity) |  |  |
| **Model 3** | | | | | |
| *Secondary No  Yes* | 43%  57% | 0.000  -3.201 | --  0.01 | 0.000  -0.831 | --  0.44 |
| *Secondary & No ViñedoViejo Yes* | 60%  40% | 0.000  0.932 | --  0.40 | 0.000  -0.326 | --  0.76 |
| *Age 16-18  19-24  25-29* | 27%  67%  6% | 3.248  0.000  1.136 | 0.00  --  0.49 |  |  |
| *Health No problems Yes* | --  -- | --  -- | (omitted because of collinearity) |  |  |
| **Model 4** |  |  |  |  |  |
| *Secondary No  Yes* | 43%  57% | 0.000  -2.886 | --  0.00 | 0.000  -2.505 | --  0.01 |
| *Secondary & No Student Yes* | 69%  31% | 0.000  1.643 | --  0.24 | 0.000  2.833 | --  0.02 |
| *Secondary & Student & No ViñedoViejo Yes* | 84%  16% | 0.000  0.116 | --  0.93 | 0.000  -0.269 | --  0.84 |
| *Age 16-18  19-24  25-29* | 27%  67%  6% | 2.010  0.000  1.463 | 0.08  --  0.37 |  |  |
| *Health No problems Yes* | --  -- | --  -- | (omitted because of collinearity) |  |  |

Table S10: Regression coefficients of educational level among young men living in Casablanca (n=122): models for total

| Explanatory variables | Prob | Initial model | | Final model | |
| --- | --- | --- | --- | --- | --- |
|  |  | Regression coefficient | p-value | Regression coefficient | p-value |
| **Model 1** | | | | | |
| *Secondary No  Yes* | 43%  57% | 0.000  -2.919 | --  0.31 | 0.000  -1.950 | --  0.42 |
| *Age 16-18  19-24  25-29* | 27%  67%  6% | 2.103  0.000  -4.437 | 0.52  --  0.40 |  |  |
| *Health No problems Yes* | --  -- | --  -- | (omitted because of collinearity) |  |  |
| **Model 2** | | | | | |
| *Secondary No  Yes* | 43%  57% | 0.000  -4.751 | --  0.12 | 0.000  -5.221 | --  0.08 |
| *Secondary & No Student Yes* | 69%  31% | 0.000  6.568 | --  0.09 | 0.000  6.100 | --  0.06 |
| *Age 16-18  19-24  25-29* | 27%  67%  6% | -1.543  0.000  -3.327 | 0.69  ---  0.52 |  |  |
| *Health No problems Yes* | --  -- | --  -- | (omitted because of collinearity) |  |  |
| **Model 3** | | | | | |
| *Secondary No  Yes* | 43%  57% | 0.000  -4.431 | --  0.31 | 0.000  -2.093 | --  0.54 |
| *Secondary & No ViñedoViejo Yes* | 60%  40% | 0.000  1.757 | --  0.64 | 0.000  0.205 | --  0.95 |
| *Age 16-18  19-24  25-29* | 27%  67%  6% | 2.728  0.000  -4.585 | 0.44  --  0.38 |  |  |
| *Health No problems Yes* | --  -- | --  -- | (omitted because of collinearity) |  |  |
| **Model 4** |  |  |  |  |  |
| *Secondary No  Yes* | 43%  57% | 0.000  -4.817 | --  0.11 | 0.000  -5.221 | --  0.08 |
| *Secondary & No Student Yes* | 69%  31% | 0.000  5.077 | --  0.28 | 0.000  4.715 | --  0.23 |
| *Secondary & Student & No ViñedoViejo Yes* | 84%  16% | 0.000  2.465 | --  0.58 | 0.000  2.696 | --  0.54 |
| *Age 16-18  19-24  25-29* | 27%  67%  6% | -1.141  0.000  -3.287 | 0.77  --  0.53 |  |  |
| *Health No problems Yes* | --  -- | --  -- | (omitted because of collinearity) |  |  |
